# Viral Gastroenteritis in Mongolian Infants

**DOI:** 10.3201/eid1101.040337

**Published:** 2005-01

**Authors:** Grant Stuart Hansman, Minako Kuramitsu, Chushi Kuroiwa, Hiromu Yoshida, Kazuhiko Katayama, Naokazu Takeda, Hiroshi Ushijima, Gungaa Surenkhand, Dugerjav Gantulga

**Affiliations:** * University of Tokyo, Tokyo, Japan; † National Institute of Infectious Diseases, Tokyo, Japan; ‡ Ministry of Health, Ulaanbaatar, Mongolia

**Keywords:** letter, gastroenteritis, norovirus, sapovirus

**To the Editor**: Viral agents of gastroenteritis affect millions of persons of all ages worldwide (*1*). The major agents include rotavirus, norovirus, sapovirus, astrovirus, and enteric adenovirus. Rotavirus is the most frequent cause of acute sporadic childhood gastroenteritis (*1*), whereas norovirus infects both adults and children and is mainly associated with outbreaks of acute gastroenteritis (*1*). These viruses are transmitted by foodborne, person-to-person, fecal-oral, and environmental routes.

In 1999, the infant death rate was 37.3 per 1,000 live births in Mongolia (*2*). Bacterial pathogens, such as Shigella flexneri and Salmonella, are commonly detected in hospitalized patients with gastroenteritis, but no data exist concerning viral agents of gastroenteritis in hospitalized patients or in the general community (*2*).

This preliminary community-based molecular epidemiologic study was the first to report viral agents of gastroenteritis in Mongolian infants. Stool specimens collected from July to August 2003 from 36 infants belonging to 25 different households from 2 areas in Mongolia were screened for rotavirus, norovirus, sapovirus, astrovirus, and adenovirus. The 2 areas were Tov Province, which included Zuun Mod (provincial center) and Bayanchandmani (provincial district center), and Ulaanbaatar area (capital city), which included Chingeltei, Bayangol, Songinokharikhan, and Bayanzurkh. A total of 48 stool specimens, which were randomly selected from negative-enterovirus specimens (poliovirus and nonpolio enterovirus (Minako Kuroiwa; unpub. data), were screened. Of the 36 infants in the study, 2 specimens were collected 3 weeks apart from each of 12 infants, and 1 specimen was collected from each of 24 infants. In 10 households, specimens were collected from 2 or 3 siblings. Clinical symptoms were recorded when available.

RNA extraction, cDNA synthesis, and polymerase chain reaction (PCR) were performed as described elsewhere (*3*); for norovirus genogroup (G) I (GI), PCR, G1SKF, and G1SKR primers were used, and for norovirus GII PCR, G2SKF, and G2SKR primers were used (*4*). For sapovirus, a nested PCR approach was used for all human genogroups (*5*). For the first sapovirus PCR, SV-F11 and SV-R1 primers were used, while for the nested PCR, SV-F21, and SV-R2 primers were used. For astrovirus PCR, Mon244, and 82b primers were used (*6*). All PCR products were analyzed by 2% agarose gel electrophoresis and visualized by ethidium bromide staining. For rotavirus and adenovirus screening, a rapid dry-spot latex agglutination test, Diarlex Rota-Adeno (Orion Diagnostica, Espo, Finland) was used.

Reverse transcription (RT)–PCR products were excised from the gel and purified by the QIAquick gel extraction kit (Qiagen, Hilden, Germany). Nucleotide sequences were prepared with the terminator cycle sequence kit (version 3.1) and determined with the ABI 3100 avant sequencer (Applied Biosystems, Foster City, CA, USA). Nucleotide sequences were aligned with Clustal X and the distances were calculated by Kimura’s 2-parameter method (*3*). The nucleotide sequence data determined in this study have been deposited in GenBank under accession no. AY590250–AY590262.

Specimens from 12 (33%) of 36 infants were positive for viral agents of gastroenteritis. Specimens from 9 infants were positive for noroviruses, specimens from 2 infants were positive for astroviruses, and a specimen from 1 infant was positive for sapovirus. All specimens were negative for rotavirus and adenovirus. Ten isolated norovirus sequences (9 persons) were classified according to the recent capsid-based sequence scheme of Kageyama et al. (*7*). Two norovirus sequences belonged to genogroup I/genotype 11 (G1/11), 4 sequences belonged to GII/3, 1 sequence belonged to GII/7, and 3 sequences belonged to GII/6 (Table).

**Table:** Mongolian infants positive for viral agents of gastroenteritis

Virus	Genogroup/genotype	Specimen*	Symptom†	Age (mo.)	Sex
Norovirus	GI/11	213-3‡	NA	4	F
Norovirus	GI/11	214-3‡	NA	24	F
Norovirus	GII/6	101-1	None	5	F
Norovirus	GII/3	109-1	Diarrhea	6	F
Norovirus	GII/6	205-3	NA	5	F
Norovirus	GII/3	209-1	Diarrhea	3	M
Norovirus	GII/3	317-1§	NA	24	M
Norovirus	GII/6	613-1	None	5	M
Norovirus	GII/7	613-3	NA	5	M
Norovirus	GII/3	609-3§	NA	5	M
Astrovirus	GI¶	121-3	NA	4	M
Astrovirus	GI	201-3	NA	5	M
Sapovirus	GI	217-1	Diarrhea	1	F

*First 3 numbers before the hyphen refer to the infant; number after the hyphen refers to the week the specimen was collected. †NA, not available. ‡Two siblings from the same household. §Only 1 of the siblings from this household was infected. ¶Astrovirus GI = serotype 1.

In 1 household, 2 female infants (isolates 213-3 and 214-3, respectively) were infected with a norovirus GI/11 strain that shared 100% nucleotide identity. This strain was likely the same and suggests a common source of contamination or person-to-person transmission. Strains belonging to this new genotype have only been detected in Japan and Switzerland (*7*).

In a different household, 2 different norovirus strains were detected 3 weeks apart in a 5-month-old male infant (isolates 613-1 and 613-3, respectively). These 2 isolated norovirus sequences shared 77.5% nucleotide identity and clustered into two different genotypes, GII/6 (isolate 613-1) and GII/7 (isolate 613-3). In spite of this infection, the infant had no symptoms of gastroenteritis during excretion of the first norovirus strain.

In 4 other households, 4 infants (isolates 109-1, 609-3, 317-1, and 209-1) were infected with norovirus strains belonging to GII/3. These 4 isolated sequences shared >98% nucleotide identity to Arg320 sequence (AF190817), which was previously found to be a recombinant norovirus (*8*). This result suggests these 4 strains are also recombinant noroviruses, though further sequence analyses of other genetic regions are needed to confirm this result.

Astrovirus was detected in 2 male infants from different households. One infant was 4 months of age (isolate 121-3), and the other infant was 5 months of age (isolate 201-3). These 2 isolated astrovirus sequences had 100% nucleotide identity, which suggests a common source of contamination. These isolated astrovirus sequences shared 98% nucleotide identity to astrovirus Oxford virus sequence (genogroup I). Sapovirus was detected in 1 stool specimen (isolate 217-1) from a 1-year-old female with diarrhea. The isolated sapovirus sequence shared 98% nucleotide identity to sapovirus Manchester virus sequence (genogroup I). Rotavirus and adenovirus were not detected in any of these specimens; further studies, including those of hospitalized infants, may be useful since infants with rotavirus infections are commonly admitted to hospitals (*9*).

Our preliminary findings have shown that norovirus was a common agent of gastroenteritis (9 of 36 persons) in Mongolian infants. In a recent report on norovirus gastroenteritis, the risk of contracting gastroenteritis was high when another household member was infected and slightly higher when that member was a child (*10*). In our study, we found 2 siblings infected with an identical norovirus strain during the same period. In Mongolia, diarrhea has become a major healthcare problem (*2*), therefore, general education in sanitation and hygiene practices may help reduce the transmission of these viruses and lessen the frequency of this disease.
